# Plant-Based Diet Indices and Depression in University Students: The Nuts4Brain-Z Study

**DOI:** 10.3390/nu18122018

**Published:** 2026-06-21

**Authors:** Valentina Díaz-Goñi, Fernando Peral-Martínez, Tomás Olivo-Martins-de-Passos, María Eugenia Visier-Alfonso, Nuria Beneit, Estela Jiménez-López, Arthur Eumann Mesas, Bruno Bizzozero-Peroni

**Affiliations:** 1 Health and Social Research Center, Universidad de Castilla-La Mancha, 16071 Cuenca, Spain; valentina.diaz@uclm.es (V.D.-G.); fernando.peral@uclm.es (F.P.-M.); mariaeugenia.visier@uclm.es (M.E.V.-A.); profesor.nbeneit@uclm.es (N.B.); estela.jimenezlopez@uclm.es (E.J.-L.); arthur.emesas@uclm.es (A.E.M.); bruno.bizzozero@uclm.es (B.B.-P.); 2 Center for Biomedical Research Network in Mental Health (CIBERSAM), Instituto de Salud Carlos III, 28029 Madrid, Spain; 3 Higher Institute of Physical Education, Universidad de la República, Maldonado 20000, Uruguay; 4 Aging Research Center, Department of Neurobiology, Care Sciences and Society, Karolinska Institutet and Stockholm University, 17165 Stockholm, Sweden

**Keywords:** diet, lifestyle, depressive symptoms, college students, young adult

## Abstract

**Background/Objectives**: Evidence on the associations between adherence to different plant-based diet indices and depression in young adults remains limited. This study aimed to analyze the associations of overall, healthy, and unhealthy plant-based diet indices with depressive symptoms in university students. **Methods**: A cross-sectional study was conducted in 2023 with self-reported data from university students in Cuenca, Spain. Adherence to the overall plant-based diet index (PDI) and to the healthy (hPDI) and unhealthy (uPDI) plant-based diet indices were calculated using data from a 137-item food-frequency questionnaire. Mild-to-severe depression was defined as a Beck Depression Inventory II score > 13 points. Linear and logistic regression models were adjusted for sociodemographic and lifestyle-related confounders. **Results**: A total of 392 students (mean age: 20.9 ± 2.4 years; 70.4% female) were included. The prevalence of mild-to-severe depression was 36.0%. Higher hPDI and overall PDI scores were associated with lower depressive symptom scores, whereas uPDI scores showed a positive but non-significant association after full adjustment. In logistic regression analyses, high adherence to the hPDI was associated with lower odds of mild-to-severe depression (OR = 0.51; 95% CI: 0.28–0.95; *p*-for-trend = 0.030). In contrast, higher uPDI adherence was associated with greater odds of depression, although the association was attenuated after adjustment for lifestyle-related variables. **Conclusions**: Greater adherence to a healthy plant-based diet was associated with lower depressive symptoms and lower odds of mild-to-severe depression among university students. These findings highlight the importance of plant food quality, rather than plant-based diets per se, in relation to depression in young adults.

## 1. Introduction

Depression is a major global public health concern, affecting an estimated 4% of the world’s population [1,2] and 6.5% of the European population [3]. In 2021, major depressive disorder ranked among the most prevalent mental health conditions worldwide [2]. Notably, approximately three-quarters of depression cases have their onset during youth [4], which corresponds with the high prevalence observed among young adults aged 18–25 years [5]. For those pursuing higher education, the transition to university represents a critical developmental period [6] marked by academic, social, and personal challenges that may adversely affect mental health [7,8,9]. Accordingly, a meta-analysis of 59 studies estimated a pooled depression prevalence of 33.6% among university students [10], highlighting the importance of addressing mental health during this life stage as a key strategy for prevention and early intervention [11].

Adherence to a healthy lifestyle has been consistently associated with better mental health [12,13,14] and reduced depressive symptoms [15]. Among lifestyle behaviors, diet has been widely recognized as a modifiable determinant of mental health promotion [14,16] and depression prevention [17,18]. In this sense, plant-based diets, characterized by higher consumption of plant-derived foods and lower consumption of animal-derived foods [19,20,21], have been suggested to confer beneficial effects on mental health outcomes [22] and depressive symptoms [23]. However, plant-based diet quality may vary according to the predominant plant food subgroups, and this may impact their health effects. This is particularly relevant when examining the association between plant-based diets and populations with suboptimal dietary habits, such as Spanish youth [24] and university students [25,26]. While a healthy plant-based diet includes mainly fresh and minimally processed plant foods (e.g., fruits, vegetables, whole grains, nuts), a less healthy plant-based diet includes high consumption of processed and ultra-processed foods (e.g., refined grains, sweetened carbonated beverages, salty snacks, sweets), characterized by its elevated levels of saturated fats, trans fats, sugars, sodium, and refined starches [27], and alcohol [28]. In this context, the plant-based diet index (PDI) makes it possible to assess the degree of adherence to dietary patterns characterized by a high consumption of plant-based foods. Furthermore, this index allows for a distinction to be made between the quality of such diets, differentiating between the healthy plant-based diet index (hPDI) and the unhealthy plant-based diet index (uPDI) [21,29].

The association between these three indices and depression has been previously explored. In a meta-analysis of 23 studies conducted on adult populations, the authors reported that greater adherence to the hPDI was associated with a lower likelihood and risk of depression [30]. However, evidence in young adults remains limited, as only two of these studies included participants under 35 years of age, and both focused exclusively on Iranian women [31,32]. Furthermore, only one of them examined outcomes related to depression [32]. For this reason, it is important to expand the empirical evidence on European young populations, especially regarding depression-related outcomes. Therefore, the objective of this study was to examine the associations between adherence to plant-based diets, differentiating between healthy and unhealthy patterns, and depression among Spanish university students.

## 2. Materials and Methods

This cross-sectional study is based on data obtained from a sample of students in various degree programs, including nursing, social work, audiovisual communication, tourism, business administration, law, education, and engineering, at the *Universidad de Castilla*-*La Mancha* (UCLM) in Cuenca, Spain, during the 2023–2024 academic year. The data analyzed are drawn from the Nuts4Brain-Z study, which was designed to estimate the associations between nut consumption and mental health outcomes in the target population. The sample size was calculated with GRANMO software (version 7.12) [33], which estimated a minimum sample size of 454 students to detect a mean difference of 0.3 units (standard deviation [SD] 2.04) on the Center for Epidemiological Studies Depression Scale-Revised [34], with an alpha error of 0.05 and a statistical power of 0.8. Considering a nonresponse rate of 20%, the total sample size was calculated at 544 students. Taking as a sampling frame the list of students enrolled in the UCLM courses (academic degrees in education, engineering, social sciences and nursing), 463 agreed to participate and met the following inclusion criterion: being regular university students at the Cuenca campus of UCLM. After excluding individuals with missing data on key variables and with implausible energy intake values, a total of 392 university students were included in this analysis (84.7%).

The data collection process was conducted between September and December of 2023. All participants were required to attend scheduled visits at the MOVI Fitness Laboratory, which is located within the Faculty of Nursing of Cuenca, Spain. Assessments were conducted between 8:00 a.m. and 11:30 a.m., Monday to Friday. The study protocol was approved by the Clinical Research Ethics Committee of the *Hospital Virgen de la Luz* of Cuenca (2023/PI1323) and conducted in accordance with the principles of the Declaration of Helsinki [35]. Written informed consent was obtained from all participants. This cross-sectional study was reported according to the Strengthening the Reporting of Observational Studies in Epidemiology guidelines [36].

### 2.1. Study Variables

#### 2.1.1. Exposure

A 137-item food frequency questionnaire (FFQ) was used to estimate food consumption [37]. The FFQ comprised 137 food items grouped into nine major categories: dairy products; eggs, meat, and fish; vegetables and leafy greens; fruits; legumes and cereals; oils and fats; pastries and baked goods; miscellaneous foods; and beverages. For each item, participants reported their usual intake using one of nine frequency categories, ranging from “never or almost never” to “more than six times per day”. Dietary intake was quantified by combining the reported consumption frequency with the standard portion size assigned to each food item in the questionnaire, based on data from the Spanish Food Composition Database [38]. The plant-based diet indices were calculated in accordance with the method originally proposed by Satija et al. [21] which comprises three indices: the overall PDI, the hPDI, and the uPDI. Prior to the creation of the indices, the foods in the FFQ were divided into 18 food groups, which were in turn subdivided into three categories: (i) healthy plant food groups, including fruits, vegetables, whole grains, nuts, legumes, vegetable oils and tea/coffee; (ii) less healthy plant food groups, encompassing fruit juices, refined grains, potatoes, sugar-sweetened beverages and sweets/desserts; and (iii) animal food groups, containing animal fats, dairy, eggs, fish/seafood, meat (red and white meat) and miscellaneous animal-based foods. The specific foods included in each food group are presented in Table S1.

For the creation of the three indices, the 18 food groups were classified into quintiles based on daily intake in grams. Depending on the index and the food category, a standard or inverse scoring method was applied. For positively scored components, the lowest quintile received 1 point and the highest, 5 points; for inversely scored components, this scoring was reversed (quintile 1 scored 5 and quintile 5 scored 1). In the PDI, plant-based food groups were scored positively, while animal-based food groups were scored inversely. In the hPDI, healthy plant-based foods received positive scores, while less healthy plant-based foods and animal-based foods were scored inversely. In the uPDI, less healthy plant-based foods were scored positively, while healthy plant-based foods and animal-based foods were scored inversely. To calculate each index, the score for each food group was summed, with a range of 18 to 90 points.

Higher scores on all PDIs indicate lower consumption of animal-based foods. Additionally, higher scores on the overall PDI indicate higher consumption of both healthy and less healthy plant-based food groups. Higher scores on the hPDI indicate higher consumption of healthy plant-based foods and lower consumption of less healthy plant-based foods. Higher scores on the uPDI indicate higher consumption of less healthy plant-based foods and lower consumption of healthy plant-based foods.

Once the indices were calculated, they were classified into tertiles to categorize participants according to their level of adherence (tertile 1: low adherence; tertile 2: moderate adherence; tertile 3: high adherence).

#### 2.1.2. Outcome

Depressive symptom severity was evaluated with the Spanish version [39] of the Beck Depression Inventory II (BDI-II) [40], a widely self-reported measure used in both scientific research and clinical practice. It has strong construct and criterion validity among university students [41]. The BDI-II includes 21 items that assess a broad range of cognitive, emotional, behavioral, and somatic symptoms of depression including feelings of sadness, pessimism, guilt, worthlessness, and self-dislike; self-critical thoughts and perceived failure; diminished pleasure, interest, and sexual desire; indecisiveness, agitation, irritability, crying, and concentration difficulties; as well as physical symptoms such as fatigue, low energy, sleep and appetite disturbances, and suicidal thoughts or wishes. Each item has four response options and is measured on a scale of 0–3, with total scores ranging from 0 to 63. Higher scores indicate greater severity of depressive symptoms, and the BDI-II total score can be classified as absent or minimal (0–13), mild (14–19), moderate (20–28), or severe (29–63) [40]. For the present analysis, mild-to-severe depression was defined as a binary outcome, with a cut-off score of greater than 13 points on the BDI-II [42,43].

#### 2.1.3. Covariates

Sociodemographic and lifestyle-related variables were included as potential confounders given the established evidence supporting their relationships with both diet and depression [44,45,46,47]. The sociodemographic data were self-reported and included age (years), sex (female, male), and socioeconomic status based on parental educational level and occupation (medium-high, low). The participants’ weight (kg) and height (cm) were objectively measured in accordance with standardized procedures, and their body mass index (BMI) was calculated (kg/m^2^) to classify the students as overweight or obese (≥25 kg/m^2^) or not overweight or obese (<25 kg/m^2^).

For lifestyle behaviors, participants were first asked about their tobacco and alcohol use over the past 30 days. Those who answered “daily” for smoking and “weekly” or “daily” for drinking alcohol were considered as tobacco smokers and alcohol drinkers, respectively. Physical activity levels were assessed via the International Physical Activity Questionnaire [48], with participants classified as moderate to high (i.e., energy expenditure ≥ 600 MET-min/week) or low physical activity (i.e., energy expenditure < 600 MET-min/week) [49]. Sleep quality over the last month was assessed using the Pittsburgh Sleep Quality Index (PSQI) [50]. Scores can range from 0 to 21, and increasing scores are indicative of lower overall sleep quality. The PSQI score was utilized to categorize the participants as having either good (score ≤ 5) or poor (score > 5) sleep quality [50,51].

Finally, the total energy intake of participants was estimated based on the responses to the 137-item FFQ and calculated in total calories per day using the updated values from the Spanish Food Composition Database [37,38]. We excluded participants with implausible total energy intake values (<500 or >5000 kcal/day) [52] and those with missing data for any covariate included in the multivariable models.

### 2.2. Statistical Analysis

First, baseline characteristics of the study participants were examined overall and according to depression status (i.e., absent or minimal vs. mild-to-severe). Categorical data were presented as frequencies and percentages. For continuous variables, means and SD were reported for normally distributed data, whereas medians and interquartile ranges (Q1–Q3) were used when distributional assumptions were not met. Normality was assessed using visual and analytical methods, including normal probability plots and the Kolmogorov–Smirnov test. Variables with a nonnormal distribution were analyzed using the Mann–Whitney U test. Categorical variables were compared using the chi-square test. In addition, the characteristics of the study sample and the mean consumption of food groups were examined according to tertiles of adherence (i.e., low, moderate, and high) to the plant-based diet indices.

Second, linear regression analyses were conducted to estimate B coefficients and their 95% confidence intervals (95% CIs) for depressive symptom scores associated with a 1-point increase in the plant-based diet indices. Scatter plots with fitted regression lines were generated to further illustrate the associations observed in the regression analyses. Binary logistic regression models were employed to estimate the odds ratios (ORs) and their 95% CIs for mild-to-severe depression (vs. absent or minimal depression) according to plant-based diet adherence tertiles (2nd or moderate and 3rd or high vs. 1st tertile or low adherence [reference category]). For both analyses, four models were estimated to assess the influence of potential confounders: Model 0: unadjusted; Model 1: adjusted for age, sex, and socioeconomic status; Model 2: Model 1 plus BMI; Model 3: Model 2 plus tobacco smoking, alcohol drinking, physical activity level, sleep quality, and total energy intake. To evaluate potential multicollinearity among covariates included in the fully adjusted model, variance inflation factors (VIFs) were calculated. Multicollinearity diagnostics indicated low VIF values for all covariates included in Model 3 (range: 1.05–1.20).

For the statistical analyses, covariates were categorized as follows: age (as a continuous variable in years), sex (female or male), socioeconomic status (low or medium/high), tobacco smoking (yes/no), alcohol drinking (yes/no), physical activity level (low or moderate/high), sleep quality (poor [PSQI score > 5] vs. good [PSQI score ≤ 5]), total energy intake (tertiles of the sample distribution) and BMI (≥25 kg/m^2^ vs. <25 kg/m^2^). Tests for linear trends (*p*-for-trend) across tertiles were conducted by modeling the median value of each category as a continuous variable in logistic regression models. To address potential concerns regarding statistical power, post hoc power analyses were conducted using the final analytical sample (*n* = 392). The statistical power of the linear and logistic regression analyses involving the plant-based diet indices was estimated separately.

Third, potential interactions between plant-based diet indices and all covariates included in Model 3 were assessed by adding interaction terms to the linear regression models. Significant interactions (*p* < 0.05) were further explored through stratified analyses.

Finally, sensitivity analyses were conducted by excluding (i) participants with moderate-to-severe depression (BDI-II score ≥ 20) and/or self-reported treatment of depression, to minimize the potential influence of more severe depression on dietary behaviors and assess the robustness of the observed associations; and (ii) participants adhering to a vegetarian diet (i.e., without consumption of red meat, white meat, or fish), to evaluate whether the study associations were driven by individuals at the extreme end of plant-based diet adherence.

All the statistical analyses were performed using IBM SPSS Statistics, version 24.0 (IBM Corp., Armonk, NY, USA). A *p*-value < 0.05 was considered statistically significant.

## 3. Results

After excluding 51 individuals with missing data on key variables and 20 with implausible energy intake values, a total of 392 university students aged 17 to 32 years were included in the analyses (mean age 20.9 ± 2.4, 70.4% female). Table 1 presents the baseline characteristics of the study participants overall and according to depression status. The prevalence of mild-to-severe depression was 36.0% (*n* = 141), including 68 participants with mild depression and 73 with moderate-to-severe depression. Mild-to-severe depression was significantly more prevalent among females than males (41.3% vs. 23.3%), tobacco smokers than non-smokers (55.4% vs. 32.1%), participants with low physical activity levels than those with moderate-to-high levels (49.1% vs. 33.7%), individuals with poor compared to good sleep quality (52.4% vs. 17.0%), and participants with BMI ≥ 25 kg/m^2^ compared with those with BMI < 25 kg/m^2^ (46.5% vs. 32.4%; all *p*-values < 0.05).

The sample characteristics are available separately for categories of adherence to plant-based diets in Table S2. Overall, participants with higher adherence to the hPDI tended to exhibit healthier lifestyle behaviors, including higher physical activity levels, lower tobacco and alcohol use, and better sleep quality, whereas higher adherence to the uPDI was generally associated with less favorable lifestyle profiles. In addition, the mean consumption of food groups included in plant-based diets according to categories of adherence is available in Table S3. The relationships between PDI, hPDI, and uPDI scores and BDI-II scores are illustrated in Supplementary Figure S1A–C.

### 3.1. Adherence to the PDI

The PDI score ranged from 34 to 70 points. In the fully adjusted linear regression model, higher PDI scores were associated with lower depressive symptom scores (B = −0.12; 95% CI: −0.30 to −0.02; Table 2). In logistic regression analyses, adherence to the PDI was not significantly associated with mild-to-severe depression. In the fully adjusted model (Model 3), students with moderate (OR = 0.63; 95% CI: 0.36–1.12) and high (OR = 0.66; 95% CI: 0.35–1.22) adherence to the PDI showed similar odds of mild-to-severe depression compared with those with low adherence (*p*-for-trend = 0.166; Table 3).

### 3.2. Adherence to the hPDI

The hPDI score ranged from 36 to 80 points. In linear regression analyses, higher hPDI scores were associated with lower depressive symptom scores (B = −0.11; 95% CI: −0.24 to −0.02; Table 2). In logistic regression analyses, Model 1 revealed that participants with moderate and high adherence to the hPDI had lower odds of mild-to-severe depression than those with low adherence (Table 3). In the fully adjusted model (Model 3), moderate (OR = 0.43; 95% CI: 0.23–0.80) and high (OR = 0.51; 95% CI: 0.28–0.95) adherence to the hPDI were significantly associated with lower odds of mild-to-severe depression, with evidence of a significant linear trend across tertiles (*p*-for-trend = 0.030).

### 3.3. Adherence to the uPDI

The uPDI score ranged from 36 to 76 points. In linear regression analyses, higher uPDI scores were associated with higher depressive symptom scores, although this association was not statistically significant (B = 0.05; 95% CI: −0.06, 0.18; Table 2). In logistic regression analyses, high adherence to the uPDI was associated with greater odds of mild-to-severe depression in the unadjusted model (Model 0; OR = 1.81, 95% CI: 1.09–3.02), the sociodemographic adjusted model (Model 1; OR = 1.78, 95% CI: 1.06–3.00) and the model additionally adjusted for BMI (Model 2; OR = 1.72, 95% CI: 1.01–2.91). However, the association was attenuated and no longer significant after further adjustment for lifestyle-related covariates in the fully adjusted model (Model 3; OR = 1.63, 95% CI: 0.86–3.10; Table 3).

### 3.4. Post Hoc Power Analyses

Post hoc power analyses indicated variable statistical power across analyses. For the linear regression models, statistical power ranged from 24% to 35% for PDI, from 47% to 89% for hPDI, and from 37% to 50% for uPDI. For the logistic regression models, statistical power ranged from 30% to 32% for PDI, from 48% to 83% for hPDI and from 59% to 63% for uPDI, depending on the tertile comparison.

### 3.5. Interaction and Subgroup Analyses

A significant interaction was observed between the hPDI and alcohol drinking (*p*-for-interaction = 0.002). Conversely, no statistically significant interactions were identified between the plant-based diet index scores and the remaining covariates (Table S4). In exploratory stratified analyses according to alcohol drinking status, the direction of the association between hPDI and depressive symptoms was inverse in both groups. However, the association was only statistically significant among drinkers. Specifically, in the fully adjusted linear regression models, higher hPDI scores were significantly associated with lower depressive symptom scores among drinkers (B = −0.31; 95% CI: −0.48, −0.14), whereas no significant association was observed among non-drinkers (B = −0.03; 95% CI: −0.18, 0.11; Table S5). Consistently, in the fully adjusted logistic regression models, participants in the highest adherence of hPDI exhibited significantly lower odds of mild-to-severe depression among drinkers (OR = 0.18; 95% CI: 0.05–0.59), but not among non-drinkers (OR = 0.80; 95% CI: 0.37–1.72; Table S5).

### 3.6. Sensitivity Analyses

The results of the sensitivity analyses were largely consistent with the main analyses. The relationships between PDI, hPDI, and uPDI scores and BDI-II scores after excluding participants with moderate-to-severe depression and those receiving treatment for depression (*n* = 73) are illustrated in Supplementary Figure S2A–C. Notably, both moderate (OR = 2.54; 95% CI: 1.15–5.60) and high (OR = 3.50; 95% CI: 1.49–8.24) adherence to the uPDI remained significantly associated with greater odds of mild-to-severe depression in the fully adjusted model (*p*-for-trend = 0.004; Table S6).

## 4. Discussion

This study examined the cross-sectional associations between plant-based diet indices and depression among Spanish university students. One of the main findings was that the associations between plant-based dietary patterns and depression differed according to the quality of plant foods consumed. Linear regression analyses showed that higher hPDI and overall PDI scores were associated with lower depressive symptom scores, whereas uPDI scores showed a positive but non-significant association after full adjustment. Logistic regression analyses were generally consistent with these findings. Participants with the highest adherence to the hPDI showed lower odds of mild-to-severe depression, whereas higher adherence to the uPDI was associated with greater odds of mild-to-severe depression, although this association was attenuated after adjustment for lifestyle-related behaviors. Taken together, these findings highlight the importance of plant-based food quality, rather than plant-based diets per se, in relation to depressive symptoms.

Consistent with these findings, previous studies conducted in young and middle-aged Iranian women (20–50 years) have reported inverse associations between the PDI and hPDI and depressive symptoms, whereas higher adherence to the uPDI was associated with greater severity of depressive symptoms [31,32]. Similarly, our study extends these observations to a population of Spanish university students, suggesting that the quality of plant-based diets may play an important role in depression-related outcomes. Higher hPDI and overall PDI scores were associated with lower depressive symptom scores; however, only the hPDI was associated with lower odds of mild-to-severe depression. Previous prospective studies further support these observations. A cohort study of Spanish middle-aged adults reported that greater adherence to the hPDI was associated with lower depressive symptom scores [53]. Similarly, a large population-based study involving middle-aged and older adults in the United Kingdom showed that greater adherence to the hPDI was associated with lower risk of depression [54]. These findings are further supported by recent meta-analyses conducted in adult populations, predominantly including middle-aged and older adults [55].

Lifestyle-related factors may partly explain our findings. Previous evidence has shown that unhealthy lifestyle behaviors, including smoking, excessive alcohol consumption, sleep deprivation, and insufficient physical activity, are associated with poorer mental health outcomes in young adults [56]. In the present study, the association between the uPDI and depression was attenuated after adjustment for lifestyle-related factors, which suggests that behaviors such as physical activity, smoking, alcohol consumption, and sleep quality may partly influence this relationship [53]. Conversely, healthier lifestyle behaviors have been associated with lower likelihood of depressive symptoms [15]. In our study, greater adherence to the hPDI was associated with healthier lifestyle behaviors, including higher physical activity levels, better sleep quality, and lower tobacco and alcohol use. Importantly, the association between the hPDI and depression remained significant after adjustment for these variables, suggesting a potential complementary role of healthy dietary patterns and other favorable lifestyle behaviors in relation to mental health outcomes. Additionally, healthier plant-based dietary patterns have been associated with better physical fitness [57], whereas higher levels of cardiorespiratory fitness and muscular strength have consistently been associated with lower risk of depression across different adult populations [58,59]. Subgroup analyses also revealed that the inverse association between the hPDI and depressive symptoms was stronger among alcohol drinkers than among non-drinkers. One possible explanation is that non-drinkers may already exhibit a healthier overall lifestyle profile, thereby reducing the relative contribution of diet quality to mental health outcomes. In contrast, diets rich in fruits, vegetables, whole grains, and dietary fiber provide antioxidants and anti-inflammatory compounds [60] that may help mitigate the oxidative stress and inflammation associated with alcohol use [61,62], both of which have been linked to depressive symptoms [63].

Several biological mechanisms may also underlie the associations between hPDI and depression. Dietary patterns rich in healthy plant foods may modulate pathways associated with inflammation, oxidative stress, neuroplasticity, and the gut microbiota [17,60]. In this context, previous research has indicated that higher adherence to the PDI and hPDI is associated with lower inflammatory profiles, whereas greater adherence to the uPDI has been linked to increased inflammation [31]. A more mechanistic explanation for the observed associations may involve the synergistic effects of specific bioactive compounds commonly found in plant-based dietary patterns. Fruits, vegetables, legumes, nuts, whole grains, and extra-virgin olive oil provide abundant polyphenols and fermentable fibers, which can modulate the gut microbiota and enhance the production of short-chain fatty acids, particularly butyrate, propionate, and acetate [64,65]. These microbial metabolites contribute to the maintenance of intestinal barrier integrity, regulate immune responses, and attenuate systemic low-grade inflammation, including reductions in inflammatory biomarkers such as C-reactive protein, interleukin-6, and tumor necrosis factor-alpha [66]. Polyphenols may further influence the gut–brain axis through antioxidant and anti-inflammatory mechanisms, promoting a more favorable neurochemical environment and supporting the expression of brain-derived neurotrophic factor (BDNF), a key mediator of neuroplasticity, learning, and emotional regulation [64]. In addition, healthy plant-based dietary patterns may be characterized by a higher intake of omega-3-rich foods, such as nuts, seeds, and certain vegetable oils, potentially contributing to a more favorable omega-6/omega-3 fatty acid balance. Omega-3 fatty acids play important roles in neuronal membrane function, neurotransmission, and the modulation of neuro-inflammatory pathways, and have also been linked to BDNF-related signaling [67]. Similarly, other plant foods such as nuts and whole grains, which are rich in fiber, antioxidants, and bioactive compounds, have been associated with a lower likelihood of depression [68,69].

These biological mechanisms may be particularly relevant among university students, a population frequently exposed to academic stress, sleep disturbances, and unhealthy lifestyle behaviors that are associated with poorer mental health outcomes [70,71]. Such dietary exposures may contribute to alterations in gut microbiota composition and increased low-grade inflammation, potentially affecting gut–brain axis signaling and neurobiological processes involved in psychological well-being [72]. Furthermore, a systematic review suggests that plant-based dietary patterns, particularly the Mediterranean diet, exhibit high antioxidant potential, as reflected by inverse associations with biomarkers of oxidative stress, including F2-isoprostanes and malondialdehyde [73,74]. Inadequate intake of antioxidants and vitamins has also been associated with a greater risk of depression [75].

Conversely, a higher consumption of ultra-processed foods such as processed meats, sugar-sweetened beverages, packaged snacks, and ready-to-eat meals has been associated with an increased likelihood of depressive symptoms [27]. These associations may not be fully explained by nutritional composition or energy density alone, but also by additives and compounds generated during industrial processing, which can impair nutrient absorption and contribute to harmful metabolic and inflammatory processes [76]. Therefore, adherence to dietary patterns characterized by greater consumption of plant-based foods such as fruits, vegetables, whole grains, and nuts, along with lower consumption of ultra-processed foods, may represent a complementary strategy for promoting mental well-being and reducing depressive symptoms [23,30,53,77].

From a public health and epidemiological perspective, our findings do not support a general recommendation for plant-based diets per se, but rather highlight the importance of diet quality within plant-based dietary patterns. Accordingly, improving adherence to healthy plant-based diets may represent a potentially modifiable approach for promoting mental health and preventing depression among young adults, although causal relationships cannot be established from the present study.

This study has several limitations that should be acknowledged. First, due to the cross-sectional design, causal relationships cannot be established. Longitudinal cohort studies with repeated measures of both diet and depressive symptoms are needed to better evaluate the proposed associations over time. To our knowledge, no prospective studies have specifically examined the relationship between plant-based diet indices and depression among university students or young adults, although this being a critical stage for mental health prevention. Furthermore, reverse causality represents an important limitation of the present study and cannot be ruled out, as depressive symptoms may influence eating behaviors and dietary habits, potentially affecting adherence to healthy and unhealthy plant-based dietary patterns. Although sensitivity analyses excluding participants with moderate-to-severe depression and those receiving treatment for depression yielded largely similar findings, the temporal direction of the observed associations remains uncertain. Nevertheless, these findings should be interpreted within the broader body of evidence, including prospective cohort studies [53] and randomized controlled trials [78], which have suggested that higher-quality dietary patterns may contribute to better mental health outcomes and a lower risk of depression. In addition, some lifestyle-related variables included in the fully adjusted models may have acted as mediators rather than confounders. However, the cross-sectional design precluded assessment of these potential pathways. Second, dietary intake and depressive symptoms were self-reported, which may have introduced recall and social desirability bias. Participants may have under-reported foods perceived as unhealthy and over-reported foods perceived as healthy, potentially influencing the estimation of the plant-based diet indices. Although dietary intake was assessed using a previously validated FFQ and participants with implausible energy intakes were excluded to improve data quality, some degree of dietary mis-reporting and measurement error is likely unavoidable. Consequently, the observed associations may have been underestimated or otherwise affected by exposure misclassification. Furthermore, although plant-based diet indices classify foods into predefined food groups, some foods and beverages are commonly consumed together (e.g., coffee or tea with sugar or other additives). Consequently, the classification of individual food groups may not fully reflect how foods are consumed in real-life settings, which could have introduced a degree of exposure misclassification. Third, post hoc power analyses indicated limited statistical power for some comparisons. Consequently, the interpretation of null findings should be undertaken with caution. Future studies with larger sample sizes are warranted to confirm these findings and to examine potential effect modifiers with greater precision. Fourth, plant-based diet indices such as the PDI, hPDI, and uPDI are constructed differently across studies, including differences in the number of food groups and scoring criteria used, which further limits direct comparisons between findings. Finally, most participants in our study consumed some type of meat, and only five participants identified as vegetarians. Although the results remained consistent after excluding these individuals, future studies should evaluate these associations in strictly vegetarian populations to better understand the role of plant-based diet quality in relation to depressive symptoms among young adults.

## 5. Conclusions

Our findings suggest that university students with greater adherence to healthy plant-based diets were less likely to have mild-to-severe depression than those with lower adherence. In contrast, higher adherence to an unhealthy plant-based diet was associated with greater odds of mild-to-severe depression, although this association was attenuated after adjustment for lifestyle-related factors. These findings highlight the importance of plant food quality, rather than plant-based diets per se, in relation to depressive symptoms in young adults. However, given the cross-sectional design, reliance on self-reported data, and relatively small sample size, the results should be interpreted with caution. Future longitudinal studies using standardized definitions of plant-based diets and larger samples are needed to confirm these associations and better inform preventive strategies in young populations.

## Figures and Tables

**Table 1:** Characteristics of the sample according to depression status.

Characteristic	Total	Depression Status		*p*-Value
		Absent or Minimal	Mild-to-Severe ^1^	
Total sample, n (%)	392 (100.0)	251 (64.0)	141 (36.0)	
Age (years), median (Q1–Q3)	21 (19–22)	21 (19–22)	20 (19–22)	0.806
Sex, n (%)				**<0.001**
Female	276 (70.4)	162 (58.7)	114 (41.3)	
Male	116 (29.6)	89 (76.7)	27 (23.3)	
Socioeconomic status, n (%)				0.914
Low	65 (16.6)	42 (64.6)	23 (35.4)	
Medium/high	327 (83.4)	209 (63.9)	118 (36.1)	
BMI status (kg/m^2^), n (%)				**0.012**
BMI < 25	293 (74.7)	198 (67.6)	95 (32.4)	
BMI ≥ 25	99 (25.3)	53 (53.5)	46 (46.5)	
Tobacco smokers, n (%)				**<0.001**
Yes	65 (16.6)	29 (44.6)	36 (55.4)	
No	327 (83.4)	222 (67.9)	105 (32.1)	
Alcohol drinkers, n (%)				0.839
Yes	136 (34.7)	88 (64.7)	48 (35.3)	
No	256 (65.3)	163 (63.7)	93 (36.3)	
Physical activity levels, n (%)				**0.025**
Low	57 (14.5)	29 (50.9)	28 (49.1)	
Moderate/high	335 (85.5)	222 (66.3)	113 (33.7)	
Sleep quality, n (%)				**<0.001**
Poor	210 (53.6)	100 (47.6)	110 (52.4)	
Good	182 (46.4)	151 (83.0)	31 (17.0)	
Total energy intake (kcal/d), Median (Q1–Q3)	2412 (1910–3052)	2455 (1892–3014)	2367 (1918–3122)	0.914

Notes: The values are expressed as absolute frequency (n) and percentages (%) for categorical variables, and median (Q1–Q3) for continuous variables. The Mann–Whitney U test was used to compare the medians of total energy intake and age by the depression status, while the chi-square test was used accordingly for categorical variables. Bold values indicate statistical significance at a *p*-value < 0.05. ^1^ Mild-to-severe depression was defined as a BDI-II score > 13 [42,43]. A BMI of ≥25 kg/m^2^ indicates overweight or obesity. Abbreviation: BMI, body mass index.

**Table 2:** Linear regression analysis of the association between plant-based diet index scores and mild-to-severe depressive symptoms.

Plant-Based Diet Indices (Score Range)	Model 0	Model 1	Model 2	Model 3
PDI (34–70)	**−0.10** (**−0.28, −0.01**)	**−0.11** (**−0.28, −0.01**)	**−0.11** (**−0.29, −0.02**)	**−0.12** (**−0.30, −0.02**)
hPDI (36–80)	**−0.11** (**−0.25, −0.02**)	**−0.11** (**−0.24, −0.01**)	**−0.13** (**−0.26, −0.03**)	**−0.11** (**−0.24, −0.02**)
uPDI (36–76)	**0.10** (**0.00, 0.24**)	0.09 (−0.01, 0.23)	0.08 (−0.02, 0.22)	0.05 (−0.06, 0.18)

Notes: The values correspond to B coefficients (95% confidence intervals) from linear regression models. B coefficients represent the change in BDI-II score associated with a one-point increase in the corresponding plant-based diet index score (PDI, hPDI, or uPDI). Models were adjusted as follows: Model 0, unadjusted; Model 1, adjusted for age, sex and socioeconomic status; Model 2, Model 1 adjusted by BMI; Model 3, Model 2 adjusted by tobacco smoking, alcohol drinking, physical activity level, sleep quality and total energy intake. Bold values indicate statistical significance at *p*-value < 0.05. Abbreviations: hPDI, healthy plant-based diet index; PDI, plant-based diet index; uPDI, unhealthy plant-based diet index.

**Table 3:** Logistic regression of mild-to-severe depression according to the adherence to plant-based diets.

Adherence to Plant-Based Diet Indices	Total (n Cases)	Model 0	Model 1	Model 2	Model 3
PDI					
Low, T1	144 (58)	1.00	1.00	1.00	1.00
Moderate, T2	130 (41)	0.68 (0.42–1.12)	0.65 (0.39–1.08)	0.67 (0.40–1.12)	0.63 (0.36–1.12)
High, T3	118 (42)	0.82 (0.50–1.36)	0.79 (0.47–1.33)	0.78 (0.47–1.32)	0.66 (0.35–1.22)
*p*-for-trend	392 (141)	0.391	0.326	0.319	0.166
hPDI					
Low, T1	142 (64)	1.00	1.00	1.00	1.00
Moderate, T2	125 (35)	**0.47** (**0.28–0.79**)	**0.45** (**0.27–0.76**)	**0.41** (**0.24–0.70**)	**0.43** (**0.23–0.80**)
High, T3	125 (42)	0.62 (0.38–1.01)	**0.58** (**0.34–0.98**)	**0.52** (**0.31–0.89**)	**0.51** (**0.28–0.95**)
*p*-for-trend	392 (141)	**0.043**	**0.032**	**0.014**	**0.030**
uPDI					
Low, T1	138 (38)	1.00	1.00	1.00	1.00
Moderate, T2	124 (50)	**1.78** (**1.06–2.99**)	1.67 (0.98–2.81)	**1.76** (**1.03–2.99**)	1.54 (0.85–2.79)
High, T3	130 (53)	**1.81** (**1.09–3.02**)	**1.78** (**1.06–3.00**)	**1.72** (**1.01–2.91**)	1.63 (0.86–3.10)
*p*-for-trend	392 (141)	**0.023**	**0.031**	**0.044**	0.138

Notes: The values correspond to odds ratios (95% confidence intervals) from logistic regression models: Model 0, unadjusted; Model 1, adjusted for age, sex and socioeconomic status; Model 2, Model 1 adjusted by BMI; Model 3, Model 2 adjusted by tobacco smoking, alcohol drinking, physical activity level, sleep quality and total energy intake. *p*-for-trend was calculated by entering the PDI, hPDI, and uPDI tertiles as ordinal continuous variables in the models. Bold values indicate statistical significance at *p*-value < 0.05. Abbreviations: hPDI, healthy plant-based diet index; PDI, plant-based diet index; T, tertile; uPDI, unhealthy plant-based diet index.

## Data Availability

Data will be available upon reasonable request to the corresponding author.

## Supplementary Materials

The following supporting information can be downloaded at https://www.mdpi.com/article/10.3390/nu18122018/s1, Table S1: Food components included in the 18 food groups used to construct the plant-based diet indices; Table S2: Characteristics of the sample according to categories of adherence to plant-based diet indices; Table S3: Mean consumption of food groups across tertiles of adherence to plant-based diet indices; Table S4: Interaction analyses between plant-based diet indices and covariates in relation to depressive symptoms; Table S5: Stratified linear and logistic regression analyses of the association between hPDI adherence and depressive symptoms according to alcohol drinking status; Table S6: Sensitivity analyses excluding participants with moderate-to-severe depression or receiving treatment, and those adhering to a strictly vegetarian diet; Supplementary Figure S1; Scatter plots illustrating the relationships between PDI, hPDI, and uPDI scores and BDI-II depressive symptom scores; Supplementary Figure S2; Scatter plots illustrating the relationships between PDI, hPDI, and uPDI scores and depressive symptom scores after excluding participants with moderate-to-severe depression and those receiving treatment for depression.

## Abbreviations

BDI-II	Beck depression inventory II
BDNF	Brain-derived neurotrophic factor
BMI	Body mass index
CIs	Confidence intervals
FFQ	Food frequency questionnaire
hPDI	Healthy plant-based diet index
ORs	Odds ratios
PDI	Plant-Based diet index
PSQI	Pittsburgh sleep quality index
SD	Standard deviation
UCLM	*Universidad de Castilla-La Mancha*
uPDI	Unhealthy plant-based diet index
VIF	Variance inflation factor
