# Supplementary material for: Plant-Based Diet Indices and Depression in University Students: The Nuts4Brain-Z Study
Source: Nutrients. 2026 Jun 21;18(12):2018. doi: 10.3390/nu18122018 (PMC13306175; doi:10.3390/nu18122018)
Supplement: Supplementary file 1 [file nutrients-18-02018-s001.zip › nutrients-4357930-supplementary.pdf]

Supplementary Material

Plant-based diet indices and depression in university students:

the Nuts4Brain-Z study

Table S1. Food components included in the 18 food groups used to construct the plant-based diet indices.

**Plant food groups**	**Food components**
Healthy	
Whole grains	Black or whole-grain bread, oat flakes, whole meal cookies.
Fruits	Orange, grapefruit, tangerines, bananas, apples, pears, strawberries, cherries, peaches, apricots, nectarines, watermelon, melons, kiwi, grapes, plums, peaches, apricots, nectarines, watermelon, melons, kiwi, grapes, dates, dried figs, raisins, prunes.
Legumes	Lentils, beans, chickpeas, peas, lima beans, broad beans.
Nuts	Almonds, peanuts, hazelnuts, pistachios, pine nuts, walnuts.
Tea and coffee	Coffee, decaffeinated coffee, tea.
Vegetables	Chard, spinach, cabbage, cauliflower, broccoli, lettuce, endive, escarole, raw tomato, carrots, pumpkin, green beans, eggplants, zucchini, cucumbers, peppers, asparagus, others: artichokes, leek, thistle, celery, onion, garlic, snow peas, chives, mushrooms.
Vegetable oils	Olive oil, virgin olive oil, olive pomace oil, corn oil, sunflower oil, soybean oil, blend of the above.
Less healthy	
Fruit juices	Natural orange juice, natural juices from other fruits.
Potatoes	Commercial potato chips, homemade potato chips, baked or boiled potatoes.
Refined grains	White rice, cereals, white bread, pasta.
Sugar sweetened beverages	Carbonated beverages with sugar, low calorie carbonated beverages, commercial fruit juices.
Sweets and desserts	Cookies, croissants, industrial bakery, homemade pastries, chocolates, donuts, muffins, cakes, nougat, fruits in syrup, cakes.
**Animal food groups**	
Animal fats	Lard, butter.
Dairy products	Whole, semi-skimmed and skimmed milk, condensed milk, cream, milk shakes, whole or skimmed yogurt, petit suisse, cottage cheese, cream cheese, cured or semi-cured cheeses, fresh cheese, custard, flan, pudding, ice cream.
Eggs	Eggs.

Fish and seafood	White fish, blue fish, salted fish, oysters, clams, mussels, squid, octopus, cuttlefish, shrimp, prawns, canned fish and shellfish, canned fish and shellfish in oil.
Meat	Chicken or turkey with or without skin, veal, pork, lamb, rabbit, liver, ham, processed meats, pates, hamburgers, bacon.
Miscellaneous	Croquettes, soupes, mayonnaise, snacks, pizza.

Note: Foods included in this table were derived from the food frequency questionnaire.

Table S2. Characteristics of the sample according to categories of adherence to plant-based diet indices.

	Tertiles of PDI			Tertiles of hPDI			Tertiles of uPDI		
	**T1 (34–52)**	**T2 (53–58)**	**T3 (59–70)**	**T1 (36–52)**	**T2 (38–73)**	**T3 (60–80)**	**T1 (36–53)**	**T2 (54–60)**	**T3 (61–76)**
Total sample, n (%)	144 (36.7)	130 (33.2)	118 (30.1)	142 (36.2)	125 (31.9)	125 (31.9)	138 (35.2)	124 (31.6)	130 (33.2)
Plant-based diet score, M ± SD	48.0 ± 3.9	55.4 ± 1.7	62.7 ± 3.0	47.5 ± 3.9	56.3 ± 2.0	64.6 ± 4.3	48.5 ± 3.9	56.8 ± 2.0	65.1 ± 3.5
Age (years), Median (Q1 - Q3)	20.5 (19.0 - 22.0)	20.0 (19.0 - 22.0)	21.0 (20.0 - 22.0)	20.0 (19.0 - 21.0)	21.0 (19.0 - 22.0)	21.0 (20.0 - 23.0)	21.0 (19.0 - 22.0)	20.5 (19.0 - 22.0)	20.0 (19.0 - 22.0)
Sex, n (%)									
Female	98 (68.1)	94 (72.3)	84 (71.2)	99 (69.7)	89 (71.2)	88 (70.4)	90 (65.2)	94 (75.8)	92 (70.8)
Male	46 (31.9)	36 (27.7)	34 (28.8)	43 (30.3)	36 (28.8)	37 (29.6)	48 (34.8)	30 (24.2)	38 (29.2)
Socioeconomic status, n (%)									
Low	24 (16.7)	23 (17.7)	18 (15.3)	24 (16.9)	24 (19.2)	17 (13.6)	21 (15.2)	19 (15.3)	25 (19.2)
Medium/high	120 (83.3)	107 (82.3)	100 (84.7)	118 (83.1)	101 (80.8)	108 (86.4)	117 (84.8)	105 (84.7)	105 (80.8)
BMI status (kg/m^2), n (%)									
BMI ≥ 25	38 (26.4)	28 (21.5)	33 (28.0)	27 (19.0)	34 (27.2)	38 (30.4)	36 (26.1)	23 (18.5)	40 (30.8)
BMI < 25	106 (73.6)	102 (78.5)	85 (72.0)	115 (81.0)	91 (72.8)	87 (69.6)	102 (73.9)	101 (81.5)	90 (69.2)
Tobacco smokers, n (%)									
Yes	31 (21.5)	19 (14.6)	15 (12.7)	28 (19.7)	16 (12.8)	21 (16.8)	20 (14.5)	22 (17.7)	23 (17.7)
No	113 (78.5)	111 (85.4)	103 (87.3)	114 (80.3)	109 (87.2)	104 (83.2)	118 (85.5)	102 (82.3)	107 (82.3)
Alcohol drinkers, n (%)									
Yes	56 (38.9)	41 (31.5)	39 (33.1)	58 (40.8)	38 (30.4)	40 (32.0)	45 (32.6)	48 (38.7)	43 (33.1)
No	88 (61.1)	89 (68.5)	79(66.9)	84 (59.2)	87 (69.6)	85 (68.0)	93 (67.4)	76 (61.3)	87 (66.9)
Physical activity levels, n (%)									
Low	22 (15.3)	17 (13.1)	18 (15.3)	23 (16.2)	20 (16.0)	14 (11.2)	12 (8.7)	14 (11.3)	31 (23.8)
Moderate/high	122 (84.7)	113 (86.9)	100 (84.7)	119 (83.8)	105 (84.0)	111 (88.8)	126 (91.3)	110 (88.7)	99 (76.2)
Sleep quality, n (%)									
Poor	75 (52.1)	68 (52.3)	67 (56.8)	81 (57.0)	60 (48.0)	69 (55.2)	58 (42.0)	77 (62.1)	75 (57.7)
Good	69 (47.9)	62 (46.7)	51 (43.2)	61 (43.0)	65 (52.0)	56 (44.8)	80 (58.0)	47 (37.9)	55 (42.3)

Total energy intake (kcal/d), Median (Q1 - Q3)	2089.6 (1602.7 - 2637.1)	2360.1 (1941.5 - 2947.9)	2909.1 (2493.6 - 3492.6)	2788.6 (2267.7 - 3378.5)	2313.1 (1765.7 - 3202.7)	2179.3 (1637.4 - 2584.8)	2828.5 (2366.7 - 3449.2)	2425.3 (1910.2 - 3102.4)	2006.9 (1532.0 - 2473.1)

Abbreviations: BMI, body mass index; hPDI, healthy plant-based diet index; PDI, plant-based diet index; uPDI, unhealthy plant-based diet index.

Table S3. Mean consumption of food groups across tertiles of adherence to plant-based diet indices.

Plant food groups	Tertiles of PDI			Tertiles of hPDI			Tertiles of uPDI		
	T1	T2	T3	T1	T2	T3	T1	T2	T3
Healthy									
Whole grains	13.3±22.8	31.4±37.6	49.1±57.3	18.1±38.3	33.6±43.6	40.1±45.0	49.3±49.4	25.4±31.7	14.1±37.9
Fruits	213.0±161.8	287.9±195.3	464.1±260.5	273.2±191.5	320.0±240.0	352.6±255.2	422.2±250.9	290.7±185.7	219.6±199.0
Vegetables	138.2±102.2	243.1±168.3	293.7±180.2	184.0±136.7	204.6±170.9	275.7±174.2	316.1±184.6	198.8±112.4	137.6±130.1
Nuts	11.7±17.5	20.9±23.3	33.1±28.4	17.3±24.7	20.0±22.7	26.8±25.7	32.4±28.5	17.5±21.6	12.8±17.9
Legumes	18.8±13.6	29.0±24.8	40.4±29.9	28.9±27.1	28.5±24.8	28.5±22.0	38.7±29.3	27.0±21.9	19.6±17.4
Vegetable oils	10.1±12.2	15.6±14.4	20.2±16.1	12.7±13.8	15.0±15.4	17.5±14.8	19.8±15.1	15.9±16.7	8.9±9.5
Tea and coffee	29.3±36.2	44.8±49.2	64.1±60.3	36.1±44.4	41.0±48.5	58.9±56.7	65.9±56.7	40.8±48.0	26.6±36.6
Less healthy									
Fruit juices	28.8±44.7	50.9±59.5	81.3±111.6	67.7±89.0	54.3±73.8	31.6±63.4	44.9±71.2	41.6±62.3	69.3±94.3
Refined grains	66.1±46.1	83.3±50.5	105.5±71.2	97.4±64.3	89.5±58.7	62.4±42.9	88.3±55.2	82.6±60.7	79.7±59.0
Potatoes	51.3±40.5	67.0±51.2	93.6±67.0	92.4±63.1	61.5±52.2	50.9±39.1	72.6±63.4	71.9±53.0	63.2±49.1
Sweetened beverages	66.2±73.4	77.7±103.2	99.2±119.9	119.8±121.8	72.9±89.0	41.8±57.6	56.3±79.6	84.9±119.7	100.4±93.7
Sweets and desserts	53.9±63.6	59.3±40.6	72.9±51.6	87.5±67.8	57.2±39.4	35.9±29.3	54.9±43.0	69.3±67.1	60.8±48.7
**Animal food groups**									
Animal fat	0.8±1.7	0.9±1.9	0.7±1.4	1.4±2.0	0.6±1.6	0.4±1.2	0.9±1.8	0.9±1.7	0.7±1.7
Dairy products	354.7±239.2	372.6±274.4	312.2±196.7	393.6±262.2	357.4±244.2	286.3±196.6	429.1±249.9	361.4±239.4	248.6±193.7
Egg	30.9±26.1	27.4±15.2	28.5±25.8	31.9±23.9	28.3±24.2	26.5±20.1	38.0±26.8	28.0±24.0	20.3±11.0
Fish or seafood	89.8±60.6	96.8±62.2	99.1±60.8	100.8±59.6	93.0±64.2	90.3±59.7	120.0±65.8	100.3±59.0	63.1±41.3
Meat	210.9±107.1	207.3±98.0	206.2±109.7	244.1±104.1	201.2±94.0	174.8±103.8	242.1±110.9	210.3±97.8	170.6±91.7
Miscellaneous foods	42.2±28.4	40.3±30.1	42.6±31.0	58.8±33.0	38.1±25.7	25.9±16.9	38.8±29.1	44.6±31.6	42.0±28.3

Notes: Values are expressed as mean ± standard deviation (grams per day) for each food group. Abbreviations: hPDI, healthy plant-based diet index; PDI, plant-based diet index; uPDI, unhealthy plant-based diet index.

Table S4. Interaction analyses between plant-based diet indices and covariates in relation to depressive symptoms.

**Plant-based diet indices**	**PDI**	**hPDI**	**uPDI**
**Covariates**	***p*-for-interaction**		
Sex	0.317	0.551	0.644
Age	0.920	0.535	0.059
Socioeconomic status	0.976	0.386	0.105
BMI status	0.491	0.286	0.154
Tobacco use	0.317	0.117	0.876
Alcohol use	0.393	**0.002**	0.488
Physical activity levels	0.519	0.253	0.784
Sleep quality	0.086	0.285	0.265

Abbreviations: BMI: body mass index; hPDI, healthy plant-based diet index; PDI, plant-based diet index; uPDI, unhealthy plant-based diet index.

Table S5. Stratified linear and logistic regression analyses of the association between hPDI adherence and depressive symptoms according to alcohol drinking status.

	***Non-drinkers* (n=256)**		***Drinkers* (n=136)**	
**Linear Regression**	**B (95% CI)**	***p-value***	**B (95% CI)**	***p-value***
hPDI	−0.03 (−0.18, 0.11)	0.661	−0.31 (−0.48, −0.14)	<0.001
**Logistic Regression**	**OR (95% CI)**	***p-value***	**OR (95% CI)**	***p-value***
hPDI Low, T1	1.00		1.00	
hPDI Moderate, T2	0.41 (0.19–0.91)	0.029	0.43 (0.15–1.22)	0.113
hPDI High, T3	0.80 (0.37–1.72)	0.566	0.18 (0.05–0.59)	0.005

Notes: Values are presented as B coefficients (95% confidence intervals) from linear regression Model 3 and odds ratios (95% confidence intervals) from logistic regression Model 3. Model 3 was adjusted for age, sex, socioeconomic status, BMI, tobacco smoking, alcohol drinking, physical activity level, sleep quality and total energy intake. Abbreviations: CI, confidence interval; hPDI, healthy plant-based diet index; OR, odds ratio; T, tertile.

Table S6. Sensitivity analyses excluding participants with moderate-to-severe depression or receiving treatment, and those adhering to a strictly vegetarian diet.

Adherence to plant-based diets	Without moderate-to-severe depression (n = 73)		Without a vegetarian diet[1] (n = 5)	
	Total (n cases)	*OR (95% CI)*	*Total (n cases)*	*OR (95% CI)*
PDI				
Low, T1	114 (28)	1.00	143 (57)	1.00
Moderate, T2	107 (18)	0.56 (0.27–1.18)	129 (40)	0.62 (0.35–1.11)
High, T3	98 (22)	0.70 (0.32–1.53)	115 (39)	0.61 (0.33–1.15)
p-for-trend	319 (68)	0.361	387 (136)	0.113
hPDI				
Low, T1	111 (33)	1.00	140 (62)	1.00
Moderate, T2	109 (19)	**0.48 (0.24–0.99)**	124 (34)	**0.44 (0.24–0.82)**
High, T3	99 (16)	**0.36 (0.17–0.79)**	123 (40)	**0.51 (0.28–0.95)**
p-for-trend	319 (68)	**0.009**	387 (136)	**0.031**
uPDI				
Low, T1	114 (14)	1.00	136 (36)	1.00
Moderate, T2	99 (25)	**2.54 (1.15–5.60)**	122 (48)	1.58 (0.87–2.89)
High, T3	106 (29)	**3.50 (1.49–8.24)**	129 (52)	1.74 (0.91–3.35)
p-for-trend	319 (68)	**0.004**	387 (136)	0.096

Notes: Values are odds ratios and their 95% confidence intervals from the fully adjustment model (Model 3). Bold values indicate statistical significance (p-value < 0.05). [1] Participants who did not consume red meat, white meat, or fish were excluded. Abbreviations: CI: confidence intervals; hPDI, healthy plant-based diet index; OR, odds ratio; PDI, plant-based diet index; T, tertile; uPDI, unhealthy plant-based diet index.

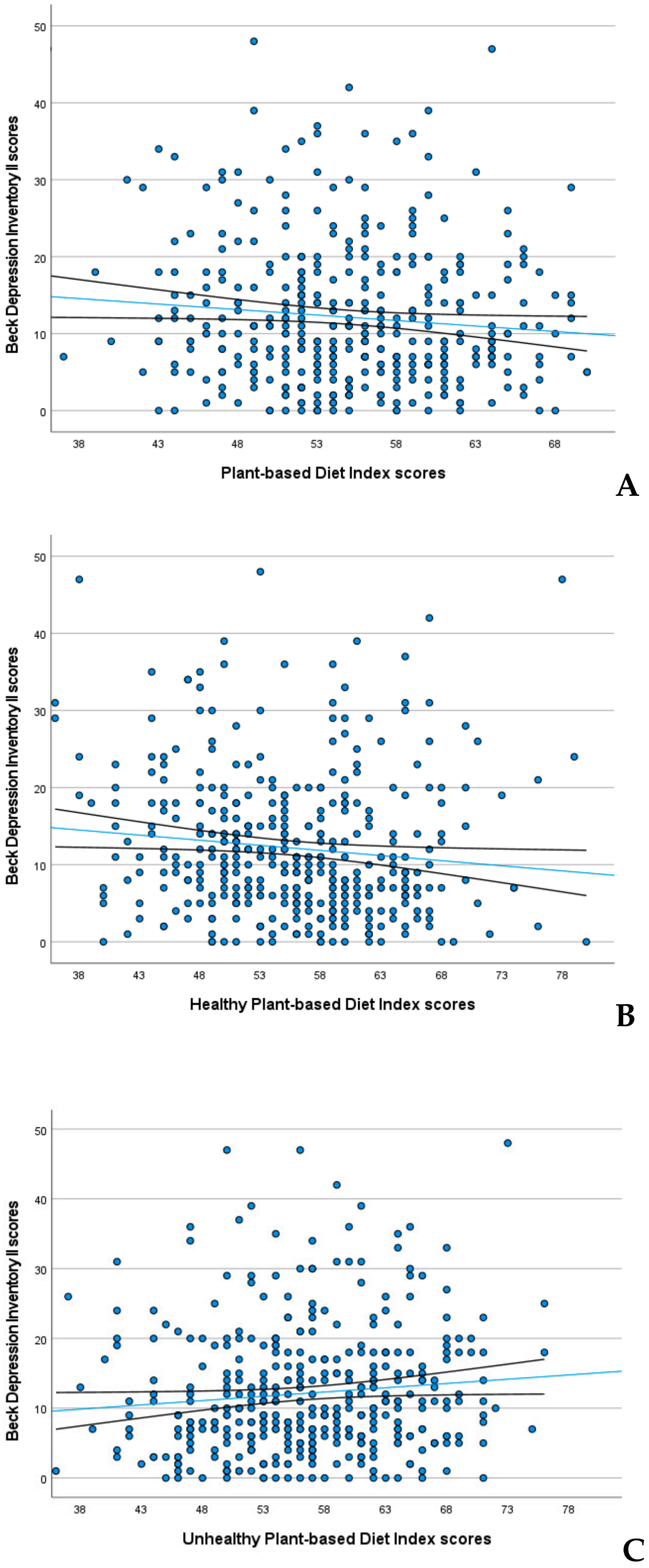

Supplementary Figure S1. Scatter plots illustrating the relationships between PDI, hPDI, and uPDI scores and BDI-II depressive symptom scores.

Notes: The blue line represents the fitted linear regression line, and the black lines represent the 95% confidence intervals.

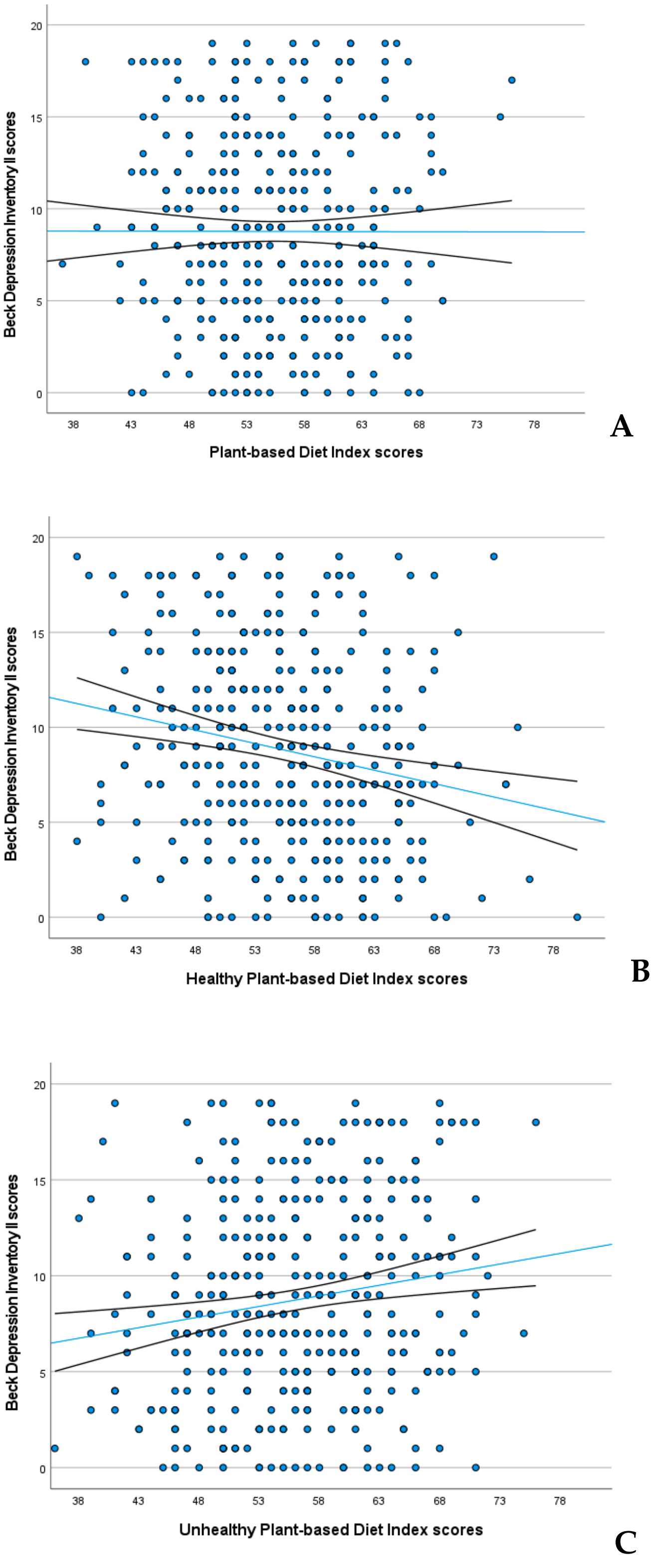

Supplementary Figure S2. Scatter plots illustrating the relationships between PDI, hPDI, and uPDI scores and depressive symptom scores after excluding participants with moderate-to-severe depression and those receiving treatment for depression.

Notes: The blue line represents the fitted linear regression line, and the black lines represent the 95% confidence intervals.
